# Motivation and genre as social action: a phenomenological perspective on academic writing

**DOI:** 10.3389/fpsyg.2023.1226571

**Published:** 2023-12-20

**Authors:** David R. Russell

**Affiliations:** Department of English, Iowa State University, Ames, IA, United States

**Keywords:** writing, motivation, genre, phenomenology, cognition, self-determination theory

## Abstract

This article discusses the relationship between motivation and genre in the context of academic writing, aiming to further bridge the gap between information-processing (IP) cognitive approaches and socio-cultural or dialogical approaches to understanding cognition. The author takes one significant recent article bridging the gap, Graham’s Writers Within Communities (WWC) model, as a starting point and attempts to add concepts from genre as social action and Deci and Ryan’s Self-Determination Theory (SDT). The article explores how genre as social action is intimately connected with motivation and how SDT’s principles of competence, autonomy, and relatedness align with the phenomenological perspective on genre and motivation. The author suggests that these theories provide a more comprehensive understanding of writing motivation, emphasizing that the perception of genre as social action is a crucial motivator for writers and that self-determination is vital to authentic self-regulation in academic writing. The article illustrates the uses of the additional theories with an interview-based case study of a dissertation writer. It ends by discussing the possible implications of this theoretical research for empirical research on student motivation from both IP cognitive and sociocultural perspectives.

## 1 Introduction

This article contributes to efforts to bridge a decades-long divide in writing studies between what have been called information-processing (IP) cognitive approaches, which view cognition primarily in terms of individual mental processes through the analogy of a computer, and socio-cultural or dialogical approaches,^1^ which view cognition primarily in socio-cultural terms and generally as an organic, embodied approach to cognition. I will focus on one aspect of cognition and socio-cultural activity: motivation.

Among several recent theoretical articles attempting to bridge the divide, Graham’s (2018) Writers Within Communities (WWC) model stands out both for its scope and for its relevance to motivation.^2^ He identifies nine characteristics of socio-cultural community(ies): purposes, members, tools, actions, written products, physical and social environments, collective history, and associated social, cultural, political, institutional, and historical forces. He then identifies four characteristics of individual members, specifically the writer(s): long-term memory resources of knowledge and beliefs, production processes, modulators, and written product. Long-term memory resources include, significantly, seven motivational beliefs. He then lays out four tenets to connect individuals and communities. Writing is simultaneously shaped by:

(1) “the community in which it takes place and the cognitive capabilities and resources of community members who create it” (p. 271)
(2) “the capacity of the writing community and the capacities possessed by members of the community” (p. 272)
(3) “variability within a writing community and individual differences in the cognitive capabilities and resources of community members” (p. 273) and
(4) “participation in writing communities and individual changes in the capabilities of community members, which interact with biological, neurological, physical, and environmental factors” (p. 274).

Because motivation is only one of many aspects of writing within communities that Graham takes up in his comprehensive theory, he does not have space to develop it beyond defining seven motivational beliefs. Aitken (2023) has persuasively argued in her chapter, “More motivating than cherry pie? The Writer(s) Within Community Model of Writing Through a Motivation Theory Lens,” that Bandura’s social cognitive theory of motivation allows us to see some broader motivational aspects of the model, and she adds minor modifications to it.

In this article, I propose further modifications to the WWC model based on two theories of motivation that the WWC model also mentions but does not develop much: the theory of genre as social action as interpreted through the lens of cultural–historical activity theory and Deci and Ryan’s Self-Determination Theory (SDT) (Ryan, 2017). These theories share common roots in existential/phenomenological thought, and they both take an organic, biological, embodied approach to cognition (Ryan and Deci, 2004). Together, they add a principled way to connect individuals and communities to understand the functioning of the writing process—how the seven motivational beliefs Graham identifies may work together to link writers and communities.

I will first outline the phenomenological approach to genre called “genre as social action” [similar to what Linell calls “communicative genres or activity types” (2009, p. 52)] to show how it incorporates motivation. Next, I describe SDT and the phenomenological assumptions it shares with genre as social action. I then illustrate their use in a case study of a dissertation student. Finally, I suggest some implications for Graham’s WWC theory and new interpretations of some findings on writing motivation from both approaches and empirically testable hypotheses to further research on student motivation using genre as social action and SDT.^3^

### 1.1 Genre as social action and the phenomenology of motivation

The theory of genre as social action has produced much work, almost exclusively qualitative, on how genres motivate and sustain writers. Based on the sociocultural and dialogic theories of Vygotsky, Bakhtin, and others, genre as social action takes a much more dynamic view of genre than IP cognitive approaches, which tend to view genres as static text types or templates, obscuring a broader contribution genre might make to L1 writing research in IP cognitive approaches.

The key difference is to see genres not as forms of words, as textual conventions, but as “forms of life,” social practices (Bazerman, 1994, p. 91). Following Carolyn Miller’s seminal article, genres are seen as “typified rhetorical actions based in recurring situations” (1984, p. 159); that is, typified responses to situations that are perceived—intersubjectively construed—as recurrent. What is recurrent is not the material situation itself (every material situation is unique) but rather our typifying perception or social construction of it as recurrent. This phenomenological view of genre is based on the phenomenological sociologist Schutz (1973), whose work lies directly behind the social constructionist tradition. Though originating in phenomenology, genre as social action has also been heavily theorized within the socio-cultural tradition in terms of Vygotsky’s developmental psychology, Luria’s cognitive psychology (through the embodied biological, not the IP approach), and, most importantly, Engeström’s activity theory, which includes explicit motive as the direction of activity [though often motives are plural and competing, as Graham notes (2018, p. 273)].

This approach to genre means, as Hidi and Boscolo (2006, p. 9) point out, that there are as many genres as there are perceived (intersubjectively construed) recurrent situations “in and out of school, whenever writing is required to express, elaborate, and communicate feelings and ideas, information and events, rules and instructions; in other words, when it makes sense to write.” Following Linell (2009) and other semiotic theorists (Prior, 2009), I would add to writing other semiotic means: speaking, gesture, semaphore, and even intra-mental communication such as self-talk.

A genre as social action is a kind of generalization or categorization of phenomenal experience that evokes—and motivates—future behavior. In Miller’s words: “A genre is a rhetorical means for mediating private intentions and social exigence; it motivates by connecting the private with the public, the singular with the recurrent,” and thus the writer with their community(ies) (1984, p. 163). In this view, people use genres as a way of perceiving/construing possible goals or directions of action. Genres help individuals and groups see “what motives one may have” (and not have) in some situation (Miller, 1984, p. 165). The theory of genre as social action accounts, in Bawarshi’s (2003) formulation, “not only for how writers articulate motives or desires but also for how writers obtain motives or desires to write—how that is, writers both invent and are invented by the genres that they write” (p. 12). With a tax form, citizens see they can pay their taxes (or protest them by turning in a ruined form); with a short answer quiz, students can show their knowledge of content; with a constitution, a group can form an institution, and so on. Bawarshi says, “To begin to write is to locate oneself within these genres, to become habituated by their typified rhetorical conventions to recognize and enact situated desires, relations, practices, and subjectivities in certain ways” and not others (2003, p. 114). He goes on to say that writing is not only a skill but a way of being and acting in the world at some point in time and space.

Genres (as social actions or activity types) are categorizations (typifications) that we create and use collectively to understand and coordinate our actions, including those involving literacy. We internalize ways of using language and other tools (including non-linguistic semiotic resources) of our physical and social surroundings, and we perceive the world through those typified modes of using tools—for example, tools for marking on surfaces, which humans do in order to write. We then interact with the world by externalizing our consciousness and enacting our feelings, thoughts, plans, aspirations, and desires, usually through typified means, such as making marks on surfaces, from cave walls to computer screens. From this perspective, perception—including the necessary typifications—constitutes the foundation of thought, reasoning, and, most importantly, language. Active perception existed prior to and is older than thought in terms of evolution. Moreover, perception precedes and provides the basis for rational and propositional thought as they evolve in humans. As Bazerman (2013) puts it, “The typifications and social-symbolic understandings that are brought to bear in the course of externalizing and internalizing meanings are strengthened” (p. 84) both in terms of embodied cognition and in terms of personal identity.

With the evolution of human languaging, we created a rhetorical world. Our perception is shaped by and shapes the oral, written, and other genres we use—genred and genreing. I use the term “genreing” on the analogy with languaging, as Linell (2009 p. 274) and others use it, to call attention to the active process of classifying, typifying, and the equally active process of perceiving, for which classification and typification are necessary (Mehlenbacher, 2019). To perceive and produce a genre is a motivated social action. When we encounter an environmental perturbation that requires a response, whether in the present or future, it is, in rhetorical terms, an exigence, which is the starting point for Miller’s (1984) theory of genre based on Bitzer’s (1968) concept of rhetorical situation.^4^ An exigence is a communicative problem in a rhetorical situation that needs solving, and when such problems are recurrent, people create genres through what Tomasello (2019) calls collective intentionality. In this way, the typified actions of writing connect the writer(s) to the collective, the community. Genre as social action connects the member(s) to the community(ies), to use Graham’s terms.

We act intentionally into the environment to perceive and respond to it—with a feedback loop that Merleau-Ponty (2013) calls “the intentional arc.” From this theoretical perspective, perception and action, conscious and non-conscious, are motivated, in that all perception and action are directed to evoke a response, feedback, from internal or external sources, or both. “From a phenomenological perspective, practical action cannot be distinguished from perception. Because people act to perceive, perception is a part of embodied action, not a passive reception that precedes or follows action” (Paul Prior, Personal correspondence, May 1, 2023). Linell (2009 p. 358ff.) makes a similar point regarding dialogic theories.

As Taylor Carman puts it in his introduction to Merleau-Ponty’s *Phenomenology of Perception* (2013), “Perception grounds the basic forms of all human experience and understanding… [P]erception is not a mode of thought; it is more basic than thought; indeed, thought rests on and presupposes perception” (p. XII). Thought is not something belonging to another realm, as in Descartes’ dualism, but rather a direct response to the perception of the world and our position within it, driven by our homeostatic and allostatic needs (Torday, 2015; Lee, 2019), which direct our attention and guide our self-regulation, as we shall see when we take up SDT concerning Graham’s WWC.

Moreover, the intentional arc suggests a profound difference between individual-based IP cognitive theories and sociocultural theories that view cognition as embodied, enactive, and embedded in the environment (Dryer and Russell, 2018). In order to act successfully, one does not need to construct a mental representation of the action on the model of a computer.^5^ One only needs to respond to feedback toward a perceived need to act into the environment (Dreyfus, 2002). This is called “next-step monitoring.” In Nagataki and Hirose’s (2007) famous example, a fielder in baseball (or cricket) does not need to mentally calculate with mental trigonometry the trajectory of the ball off the bat and triangulate the location it will land. They only need to position and reposition their body to stay between themself and the ball in the air. With much practice, they develop a “felt sense” of how to move to be in position to catch the ball.

The perception of a genre as social action may elicit a “felt sense” that one should write. As Gendlin (1982, p. 37) describes it:

A felt sense is not a mental experience but a physical one. A bodily awareness of a situation or person or event. An internal aura that encompasses everything you feel and know about the given subject at a given time—encompasses it and communicates it to you all at once rather than detail by detail. Think of it as a taste, if you like, or a great musical chord that makes you feel a powerful impact, a big round unclear feeling.

With practice, one may develop an elaborated felt sense of a genre as social action that enables highly skilled performance. However, even a novice, having experienced the felt sense, the exigence, of needing to write, can begin using next-step monitoring to feel their way forward, whether with the aid of immediate feedback, with the memory of writing previous genres in different social actions, or with myriad other resources such as instructions, models, and so on, as Graham’s theory (and others) elaborates in terms of members’ resources (p. 265).

As Hidi and Boscolo (2006, p. 2) point out, “(IP) cognitively oriented scholars view writing as interrelated processes of different levels of complexity” in accomplishing a writing task (producing text) in some task environment. I suggest here that a writing task might be viewed as a social action or communicative genre in the sense that it is a typified textual (or other semiotic) response to a recurring social situation within some stabilized-for-now social practice or activity type, such as a history book report or an essay answer in US high school history courses.^6^

Graham’s theory explicitly builds on socio-cultural theories, specifically “including activity theory and genre theory” (p. 258). WWC’s analysis of community dynamics is essentially Engeström’s activity theory structure, which he alludes to, citing Engeström’s model of expansive learning (Greeno and Engeström, 2014). Moreover, though Graham does not explicitly develop the activity theory connection between the community and individual levels, it is implied. Subject(s)/member(s)/writers(s) use tools to act on some object with some motive to achieve an outcome/written product. Indeed, Graham’s “Basic components of a writing community” diagram (p. 264) is four concentric circles. The central two circles contain four of the seven components of Engeström’s activity theory model—subject/s (writer/s), tools, object (goal/s), and outcome (written product). The three other AT components are community, which Graham includes in the outer two circles, division of labor, and rules, the latter two of which are elsewhere discussed in terms of typification.

Graham’s WIC model also clearly incorporates the phenomenological concept of typification, the phenomenological basis of the theory of genre as social action. He begins, “Actions are the typical practices that a writing community employs to achieve its writing purposes (Russell, 1997)” (2018, p. 258). He mentions “typified actions,” “typified patterns of action,” “typified practices,” or “typified patterns (routines, schemas)” some 16 times. However, Graham does not use the term genre, much less genre as social action, after its mention on the first page (258). As an addition to WWC, I suggest that the genre as social action (typified forms of words) can connect the individual writer(s) with the community(ies) with and for whom they write. The genre as social action can be seen as a nexus for understanding what motivates writers.

### 1.2 Self-determination theory and the phenomenology of motivation

Another theory of motivation that Graham briefly discusses—also based on phenomenology—might add minor modifications to the theory that further its reach and power. Actively perceiving and then writing a genre as social action is agentive; it requires not only a certain amount of but also certain kinds of motivation. One way to understand motivation in terms of genre as social action is offered by Deci and Ryan’s (2013) self-determination theory, which has been important to research on L2 writing motivation but largely ignored in L1 writing research (Graham’s recent intervention study of writers’ choice is an important exception to be discussed later).

Deci and Ryan (2013) posit three basic psychological needs that motivate humans: competence, autonomy, and relatedness. Humans are social animals and need relations with other people (i.e., community); they must interact with the world competently to survive. However, to be fully human, they must also exercise autonomy—what the tradition of existential phenomenology calls freedom. SDT research has found that, in general, positive extrinsic motivations (e.g., money, grades) have a more immediate effect but fade more quickly, while positive intrinsic motivations (relatedness, competence, autonomy—including feelings of interest and curiosity) are longer lasting and thus more powerful overall. Indeed, extrinsic motivators may have adverse effects if perceived as limiting the writer’s autonomy.

However, extrinsic and intrinsic are not a simple binary in SDT; they exist in a continuum. SDT allows partially internalizing motives through social interaction and reflective choice, making extrinsic motivators more intrinsic. SDT calls one position on the continuum identification. If one identifies strongly with others in some area of life and one appropriates or internalizes the motives of those others, one can come to feel that the motives of others are one’s own.

How can we understand the dynamic of self-determination in terms of genre as social action? Both the embodied version of phenomenology and SDT provide similar answers. Ryan and Deci (2004) argue in their comparison of existential phenomenology to SDT that there are profound similarities in the theories, all relevant to motivation in writing.

• Both emphasize that humans have autonomy—freedom to act out of one’s authentic self.
• One experiences autonomy to greater and lesser degrees in relation to one’s authentic self, depending on one’s material and social contexts and choices.
• Our performance and wellbeing improve as we perceive greater autonomy—it is motivating (and its perceived lack is demotivating).
• “Where autonomy enters the picture it is in this realm of meaning. As existentialists have argued, we act in accord with the meaning of events, and it is in the reflective construction of meanings that we can find our possibilities.” (Ryan and Deci, 2004, p. 467).
• “Although social contexts can have a clear impact on autonomy, in an ultimate sense, autonomy is something one must also cultivate within oneself and have the courage to enact. That is, in every instance one can act autonomously, which requires that one act in accord with what is authentic and real” (Ryan and Deci, 2004, p. 473).

Both SDT and genre as social action envision the relationship between members and communities as dialogic and, often, in tension. A crucial part of the writing process may be using a community’s genres until they become “one’s own” through imitation (Bandura, 1962) or anticipatory socialization (Merton, 1968). However, a member’s creative use of genres may change the community and its intentions/motivations/desires—though often with a struggle. As Bawarshi (2003) puts it:

The power of genre resides, in part, in [a] sleight of hand, in which social obligations become internalized as seemingly self-generated desires to act in certain discursive ways. This does not mean, however, that writers’ desires are completely determined, as evidenced by the fact that textual instantiations of a genre are rarely if ever exactly the same. Every time a writer writes within a genre, he or she in effect acquires, interprets, and to some extent transforms the desires that motivate it (p. 91).

Another central concept for SDT research is self-regulation. Again, Ryan and Deci (2004) point out the connection between SDT and existentialist/phenomenological theory:

• “This sense of autonomy is not simply a functionless construction, but rather it is a phenomenal state reflective of the quality of behavioral organization” (p. 474)—self-regulation, in other words.
• “Autonomy concerns how various urges, pushes, desires, primes, habits, goals, and needs from the *brain, the body, and the context* are *orchestrated* within the individual” (p. 450)—self-regulation (italics mine).
• “Behavior is experienced as autonomous when one’s actions are truly self-regulated, meaning one’s actions are self-endorsed and congruent with values, motives, and needs… rather than being controlled or entrained by forces alien to them” (p. 453).

Recent sociocultural theories, particularly embedded, embodied phenomenological approaches, similarly point to “BBE”—brain–body–environment—as a single system from which motivations arise (Varela, 1996; Thompson, 2007; Gallagher, 2012). For successful performance, thoughts, behaviors, and the environment (physical and social, direct and distal) must be orchestrated [Both Merleau-Ponty (1964, p. 54) and Schütz (1951) use the orchestra metaphor for behavior].

Both SDT and the phenomenological approaches to motivation described here recognize the deep embodied structure of self and its regulation. Humans do not naturally learn to write. Many cultures do not have writing (none had it until relatively recently in human history—roughly 5,000 out of at least 50,000 years ago) (Lieberman, 2007). Unlike speaking, writing is not embedded in human cognitive and anatomical architecture but instead built on prior functional systems, either those familiar in many other mammals (e.g., active typifying perception, memory, problem-solving action, sociality, cooperation) or prior functional systems developed in humans, such as indexing (pointing), tool making and use (especially incising or marking), and, of course, oral languaging (Hasson et al., 2018). All normal humans learn/acquire these functional systems as part of their normal development in every society, literate or not. Functional systems exist not only within the individual but also within social groupings, as theorized by Schutz (1973), Deci and Ryan (2004), and Merleau-Ponty (2013), among others, in phenomenological and socio-cultural traditions. Internal and external functional systems are mutually embedded—engaged. Indeed, “external” and “internal” only exist in relation to a highly permeable skin barrier.^7^

Not only are one’s social self, others, and the cultural tools in use (including genres) inseparable from the bodies of others but also one’s physical self, one’s living body, in that one’s body affects and is affected by the bodies of others. One’s body exists because of and in relation to other bodies from conception, if not before. We are not only intersubjective but also “intercorporeal,” as Merleau-Ponty puts it (2013). Moreover, this extends to our genres as social action. Genres not only imply structured knowledge but also structures of embodied action. As Gregersen (2011, p. 101) puts it: “We know genres and we know what to do with genres.”

Graham’s WWC briefly mentions SDT’s distinction between intrinsic and extrinsic motivation when he takes up the fourth belief about writing: motivation, “why one engages in writing” (2018, p. 256). Moreover, Aitken (2023) briefly suggests that SDT might be used to modify, in a minor way, Graham’s theory of motivation, especially with Bandura’s social cognitive theory. I suggest ways that SDT might further contribute to Graham’s theory and expand our understanding of writing motivation by employing the phenomenological perspective it shares with genre as social action. The goal is to see how motivational beliefs function together.

Graham (2018, pp. 266–267) identified seven sets of motivational beliefs (MBs) that influence writers: (1) the value and utility of writing; (2) whether or not one likes to write or views writing as an attractive task; (3) the writing competence; (4) why one engages in writing; (5) why one is or is not successful; (6) identities as writers; and (7) writing communities. All of these might be seen wholly or in large part as a function of the genre as social action.

• (4) One’s perception of the genre as social action provides the reason for writing—its initiating exigence or BBE perturbation—and thus
  ○ (1) the value and utility of writing or not writing—in terms of its potential to maintain or improve life, including:
  ○ (2) the emotional valence, positive or negative, and its degree, in comparison to other genres and social actions or in comparison to not writing, and
  ○ (3) the felt sense of writing competence for the genre, which impels (or resists) moving fingers to write along an intentional arc shaped by the genre as social action.
• (6) The genre as social action also provides the identity(ies) one can (and cannot) have as a writer of this genre as social action,
• (7) the readers/audience one can have in this genre as social action, and
• (5) the criteria for success, derived from next-step monitoring of the feedback loop of the intentional arc.

The following case study illustrates how Graham’s motivational beliefs grow out of genre as social action in one writer’s struggle for knowledge, self-determination, and self-regulation.

## 2 An illustrative case study using original data

The illustrative case study that follows uses interpretative phenomenological analysis (IPA), “a well-established qualitative approach developed to investigate individuals’ lived experiences,” as Smith and Fieldsend (2021, p. 147) put it, through an in-depth interview focused on evoking the felt sense of a specific moment.

### 2.1 Description of the case study

The following data are from an HSRB-approved study asking the general research question: What is the felt sense of slowing down or stopping writing or resuming writing after slowing or stopping? The goal is to understand not only writer’s block but also the normal processes of stopping and restarting academic writing. Participants completed two semi-structured online video-recorded interviews of about 1 h each, separated by sufficient time to have finished the writing project. In the first interview, after some questions about the writing task and situation, we asked each participant to point to “a place where you slowed down or stopped while writing something important” and evoke that specific moment. Interviewees provided some background on that text and themselves to allow us to understand their evocation of the moment in their writing and their felt sense of slowing and resuming.

I focused on one participant because her first interview (the only one discussed here) focused on her motivation, and she repeatedly mentioned the term. Moreover, she was at a point in her Ph.D. program when she had taken a short dissertation writing course (required of everyone in her program) and therefore had a vocabulary for talking about writing.

Her interview was analyzed using Nvivo. For the case study reported here, her uses of the word motivation were identified and then coded for (1) their valence (position, negative, neutral, mixed), (2) the emotions expressed around uses of the word (textually, visually, and vocally), and (3) the role she attributed to the motivations and associated emotions in stopping and restarting. The analyzed data were then interpreted (redescribed) through the terminology and constructs of both phenomenological and IP cognitive theory.

The analysis was then presented to the participant for comment.

### 2.2 The participant

Kel (pseudonym) is a Ph.D. student at a large Midwestern university, working on the pilot project she must complete before officially beginning her dissertation, a mixed-methods social science project using a survey and selected interviews. She has completed gathering data for it, drafted the methods section, started analysis of her data, and begun writing sections of the report on the pilot that she will present to her committee for approval before she can “scale it up for the full dissertation.” The interview was conducted by a student on the research team experienced in interviewing, and there was an evident rapport between the two, perhaps because Kel had also done considerable interviewing and wanted to cooperate in another interview study to get the interviewee’s perspective. My perspective as a senior scholar who had a challenging experience writing the dissertation at a problematic time gave me a particular empathy. However, it may have pushed me to draw conclusions I would not otherwise have, though I am not consciously aware of any now.

## 3 A descriptive case study

“An especially important goal of descriptive research conducted with the WWC model,” Graham says, “is to describe how the characteristics of the writing community and members’ individual differences function conjointly.” This descriptive case study illustrates how Graham’s seven motivational beliefs (MBs) function together to connect her with her communities through her perception of genre as social action. It then describes the participant’s felt sense of writing and her feelings of autonomy in terms of existential phenomenology and SDT. Finally, it illustrates self-regulation processes using genre as social action and SDT.

### 3.1 Genre as social action: orchestrating motivational beliefs

For Kel, the exigence for writing (MB 4—why she engages in writing) is the genre of the IMRD report. She must write it to get a degree, which provides its extrinsic (MB 1) value and utility. Kel reports feeling a great deal of pressure and questioning her (MB 3) *competence* to write the new genre and, with it, a whole different view of (MB 2) the attractiveness of writing: “I’ve never had negative feelings about writing. I have always been, it’s always come easy to me, and people have always complimented my writing—until I became a research writer.” She is writing a new genre as social action, an IMRD report of a pilot study leading to a dissertation based on mixed-methods empirical research. This is her task and task environment, to use IP cognitive terms.

However, there is much more going on here than learning a new set of genre conventions—forms of words. The new genre as social action implies for her a new identity as a writer (MB 6): “until I became a research writer,” she says, and entered into what Bazerman (1994, p. 91) calls a new “form of life.” Kel displays a situated sense of struggle with the typified genres and activities of her research, especially here in the sub-genre of the literature review, and complex—plural—motivational states, as she feels the pull of different (MB 7) writing communities, different sources of *relatedness*, in SDT terms. Graham critically points out that communities, identities, and motives are multiple. In this case, I want to notice how genre as social action brings that multiplicity into focus as social action.

“Lit reviews are so hard for me,” she says, because “I feel that I’m learning a whole new level of backing up claims and motivating, you know, research. It’s like a whole new approach to what I always thought came easy to me doesn’t come easy anymore.” The sub-genre of the literature review “motivates” research in that it shows why the researcher/writer and the readers (other researchers or users of the research) should move their attention in some new direction. The (MB 5) criterion for success is whether it aligns the previous knowledge in the disciplinary sub-community, the previous direction of attention, with some new knowledge claim and some new claim on their attention.

However, the literature review is also, for her at this moment, a threatening hurdle in her underlying desire to gain a doctorate, a mandatory task motivating her to address something she may have avoided or seen outside her realm previously, and she now feels must push herself through her discomfort and feelings of lack of self-confidence despite her memories of other writing tasks/environments/communities where she has felt competent. It forces her to question (MB 1) the very value and utility of writing this genre in her life.

To avoid the literature review, she returns again and again to writing and revising the data analysis and methods sections. She seeks further help from YouTube tutorials on writing literature reviews by a complex software program called Nvivo to provide, as she says, “motivation to get started on that hard, hard part for me.”

Nevertheless, she feels little motivation. Overall, she feels demotivated, like an imposter:

It felt overwhelming, and I… I mean, I know this is common for Ph.D. students, but I have these moments of, like, “I can’t do this.” I’m not, you know, it’s this imposter syndrome, like, takes over my brain.

Like, everybody’s read more than me at this point. I haven’t read enough. I haven’t done enough. I haven’t written enough. It’s… and I have to just stop that. I just have to turn that off because that’s just the devil on my shoulder.

She is, for now, a kind of imposter, pretending she is a researcher when she is not yet.

Kel immediately ties her lack of confidence and her feeling of being overwhelmed by the social action of the sub-genre to her long-term career prospects and, indeed, her future identity:

And I think definitely this process is shaping what I like, like, what career roles I have. You know, I no longer want a job focused on research. I like it, but I only like it when I have a lot of help from other people, when it’s a team, because I like to bounce ideas off people.

Her identity as a researcher is bound up with the genre and activity of the experimental article—and the dissertation it is based on (which, unlike the research article, must be individual). This is a source of anxiety and demotivation.

And I like to have other people sort of validate what I’m thinking. And, and, you know, the point of a dissertation is to establish yourself as an independent thinker and research or… so I’m not getting that feedback.

And it’s really hard for me. I’m, I’m doubting everything. So, in that moment before, and right after I (slowed and stopped writing), it was I was feeling overwhelmed.

In this crucial moment of slowing and stopping, she expressed a lack of competence so strong that she felt overwhelmed. The source of her feeling overwhelmed has to do with the institutional requirement to be, in SDT terms, an independent agent (autonomy) to get the degree (and get on with her and her family’s life) in tension with her need for connection (relatedness) with other students/researchers, a team “to bounce ideas off.”

### 3.2 The felt sense of writing and the authentic self in existential phenomenology and SDT

To understand Kel’s motivation (and lack of it), we might turn to Merleau-Ponty’s felt sense in an intentional arc and to Deci and Ryan’s concept of self-determination (2013). Both concepts focus on the conditions for agentive social action, autonomy, or freedom. Writing a new genre as social action means, at the most basic level, a felt sense of one’s competence in the task and of one’s ability to perform. However, other considerations in and around ability affect motivation, other felt senses, and motivational states. Kel, in terms of SDT’s basic needs, might be thought of as doubting her competence in this genre as social action because she is caught between the requirement that she act autonomously as a dissertation writer and her need for relatedness—the team “to bounce ideas off of” she longs for.

Moreover, the dissertation rules constrain her autonomy because she cannot collaborate with others. The felt sense of writing ability presupposes an affirmative answer to “Am I allowed to?” One may feel one could perform if one is allowed to do so in one’s own way, yet one cannot without risking adverse consequences (real or imagined) for performing the writing task (or performing it in one’s own way) beyond the moment of writing. The genre as social action of the dissertation does not allow her to collaborate with other students.

In addition, permission might imply not simply permission to write a text but permission to be (at least provisionally) one who writes this genre in this social action: identity, in other words. Writing a genre as social action involves perceiving oneself within an intersubjective community of those who write the genre, acceptance as a member of some social world, some life-world, as Schutz (1973) terms it. Her struggle with this genre as social action makes her question her identity.

Moreover, writing a genre as social action also risks a loss—ceding—of autonomy and even one’s previous identity to the group. Does one have the willingness or desire to write the task and become a member of that intersubjective community? The phrase is often: “I can bring myself to do this” (or “I can’t bring myself to do this”). As Groucho Marx reportedly said, “I don’t want to belong to any club that would accept me as one of its members” (Quote Investigator^®^, 2011 “I don’t want,”). Kel slowed down as she questioned her desire to be part of the community of researchers.

One can conclude that writing a genre (as social action) includes not only ability but also power and permission, acceptance and identity (present and future possible), desire, and identification. Kel is unsure she wants to be a “research writer” who must write this “hard part,” the research review, where she must take a personal, agentive position and assume a new identity and authority. She has a deep “interest” in writing some ways and not others—not interest as mere curiosity or attention but interest as an agentive stake in the outcome, the investment of her very self. Moreover, because the social action involves writing, making potentially permanent marks that endure across time and space, all of these motivational states are operating not only in the present moment, where the writing is happening (or meant to); they are operating potentially in the imagined future of readers responding, of life consequences, large or small.

Indeed, competing motivations lie at various places on the continuum SDT posits between extrinsic and intrinsic. Kel’s extrinsic motivations intertwine with her intrinsic motivations as she struggles with whether or not she will identify with researchers (and write their genres). Her familial and financial future rests on graduation (extrinsic motivation) and thus the subgenre she finds so hard.

She wonders if pursuing research (and its genres, with the motivational path the IMRD genre entails) is being true to herself, authentic, or a diversion. In SDT terms, she worries that extrinsic motivators may be in play and thus demotivating. There is a lack of confidence and a perceived crisis of values (MB 1). She wonders if research writing is “really” her. To be or not to be a person who writes like that and those people. Should she internalize their motives and make their genres (and social actions) hers?

From outside her perspective (more precisely, from the perspective of insiders in the field), it is pretty clear that “a job focused on research” in her field involves “a lot of help from other people,” a team where one can “bounce ideas off people.” Furthermore, she realizes that her institutional position as a Ph.D. student, writing a dissertation “to establish yourself as an independent thinker and researcher,” keeps her from “getting that feedback.” Nevertheless, she feels “overwhelmed.” Unsurprisingly, it is difficult for her to get writing again, self-regulate, to use the SDT term, or maintain the “intentional arc,” to use Merleau-Ponty’s phenomenological term.

### 3.3 BBE system of motivation and SDT self-regulation

As Kel’s comment about her lack of a “team” suggests, there are others in her BBE system of motivation here, proximal and distal. She mentions many in the interview: children, husband, officemate, dissertation director, fellow Ph.D. students, her imagined future “team” of colleagues/collaborators, the subjects of her study represented in the quantitative data, and—most saliently at this moment—authors of the literature review articles she is summarizing. As Paré (2014) says, when we look at relationships in writing, “the rhetorical situation (or task environment) suddenly becomes quite crowded” with people (p. A-9).

We now come to the phenomenological moment being specifically analyzed: Her slowing down and restarting on the literature review, and thus, to her self-regulation, her BBE system of motivation. I suggest that many of her self-regulatory behaviors proceed from the genre as social action she is attempting. Paré et al. (2007) have shown that the dissertation is a complex multi-genre, with several embedded social actions—and perforce motivations—sometimes conflicting or competing. As we have seen, she is extrinsically motivated to finish the dissertation to get a university teaching job. However, this involves writing a research genre, the IMRD, with the social action of adding new knowledge, and a sub-genre, the literature review, with the social action of describing existing knowledge other researchers have found so she can locate and claim what she is adding. She mentions various genres in the interview that regulate her behavior: APA citation style, university and department documents regulating her dissertation process, conferencing with and getting feedback from her advisor and committee members, and delivering conference papers to meet expectations, etc.

As we have seen, at that crucial moment of slowing and stopping, she needs more confidence in writing the IMRD and questions whether she is even motivated to write it. However, she knows it will be much more difficult for her to get a university teaching job without a Ph.D. and thus needs to write a dissertation. She is discouraged and lacks motivation, perhaps because it is mainly extrinsic, something she feels forced on her (external locus of control). However, the social action of the literature review sub-genre elicits self-regulatory behaviors that seem to move the locus of control toward intrinsic motivators. Recall that extrinsically motivated behaviors can become more or less intrinsically motivated as they are perceived to align with one’s own integrated values and beliefs—”integrated regulation,” in Ryan and Deci’s term (2004, p. 453). Despite her discouragement, she then behaves in ways that show her understanding of the logic of the genre expectations and her alignment with that logic—how the research subcommunity’s disciplinary expectations realized in the genre as social action can help realize her deeper motives.

Just before she slowed down, she had returned to writing summaries of articles for the literature review. She thought if she could “just get back into the reading… it’ll motivate me to write. All right.” She clearly understands that the literature review’s social action is indeed social, involving other people in the sub-community of researchers on her topic (Bazerman, 1988; Hyland, 2000) (Unlike most graduate students, she had a short course on research writing, where she was instructed on how to write the key sub-genres, such as the methods section, the results and discussion, and the literature review).

At the slow-down moment, she was in her office, before the quarantine, with another graduate student, listening to music on her headphones to relax her (corporeal self-regulation) and to minimize distractions from her officemate (intercorporeal self-regulation).

She had begun stacking printouts of the articles for her literature review—a very physical, material action, again based on the social action of the review—into two stacks. She was also classifying them on one screen into a two-column Google Doc figure based on the stacks while glancing at her data tables on another screen. Note the multi-modal self-regulation of the paper stacks and the two screens, which represented, respectively, the stacks of research articles according to the authors’ positions on the topic and the data from people she had researched—again, a physical, multi-modal configuration reflecting the social action of the literature review sub-genre in relation to the IRMD genre. She is an active agent, seeking ways to understand the literature, not simply following instructions or protocols from the regulatory documents.

However, she feels considerable frustration because she is unsure if she has correctly classified articles or, as she recalls asking herself, “Am I just placing some weird label on it that I came up with from my own, you know, for my own interpretive purposes? So that was a slowdown moment.” She feels intellectually responsible to be fair to the authors in her literature review—that is, authentic, in line with her values and those of the field, not selfishly pursuing her “own interpretive purposes” to the extent that she distorts what others “say” (wrote). Indeed, the stress she feels comes from her worry that she is acting out of inauthentic, selfish motives. As Ryan and Deci say (2004, p. 457), people can “access a direct source of knowledge concerning the degree of integrity in our own actions. Thus, when people behave, they have some internal information for judging whether the behavior is authentic or imposed, self-endorsed, or alien.” That is, in phenomenological terms, people have a felt sense of whether something is authentic, “integrated regulation.” Thus, Kel’s motive is not to avoid doing something that will violate some rule that will get her in trouble. She is attempting to get the classification of the literature right so that she is not missing something important in the community’s expectations.

She then looked down from her screens at her desk and saw “a couple of sticky notes,” large ones. She had previously put various lists of article authors on each sticky note for her literature review. She classified them with “curlicue” brackets and arrows pointing to the criteria according to which she had grouped them.

She returned to her two-column literature review figure in Google Docs and added a double-headed arrow between the columns. The arrow allowed her to create a continuum to put studies that did not fit at either extreme.

And then quickly I was like, “Oh, but there’s so many other ways to think about this research. It’s not just from the perspective of how they designed their studies. It’s from the perspective of how they interpreted results or, you know, what data they collected.”… and it was like, okay, this is helpful. This moves me in the right direction.

She perceived this as a breakthrough that motivated her to go on writing, though only briefly, as we will see.

It felt, it felt really good. It felt like, “Oh, okay. I can do this.” I can—if I can have a visual, then I—it’s not just a stack of this many research articles that I need to, you know, figure out where to put in the lit review. It’s something that I can start with the visual, and I can, I can start to figure out how they, how they work so that I can figure out where to put them in my own lit review.”

Kel borrowed the visual genre from a previous paper, as she explains: “I had just finished another paper where I envisioned a continuum of sorts…. And so that was in my head, and I thought, “Oh, this is the same. This, this is the same” (On genre borrowing, see Tardy, 2012).

Her motivation to contribute to the written conversation in the social action of the literature review returned and, with it, a shift in emotion from frustration to creative energy as she orchestrated the physical articles in the stacks, the visual representation in the on-screen Google doc, and the post-it notes (Spinuzzi, 2003) with curlicues [an occluded genre (Swales, 1996)] for organizing information flexibly) in order to self-regulate.

It was branching off of other people and how we could extend what they’ve found. And I could see it serve in the bigger picture of the literature instead of just this long list of to-do items that I am responsible for. And so that provided some new motivation.

The motivation comes from the meaningful conversation (written) that the genre’s social action demands.

However, the new motivation and new start were only a part of the long dissertation writing process. Other motivations followed, other slow-downs, stops, and restarts.

And then, I think very quickly after that, it was like, time for me to go, and I had to pack everything up and go home. And once I get home, I don’t make much progress because I have kids. So the kids are, you know, they just take over once I get home.

However, she later used the post-it-inspired continuum visual to produce an outline of the literature review. Furthermore, she did finish the dissertation and graduated. However, that is another analysis, based on the second interview.

To summarize, the social actions of the IMRD genre and research review sub-genre elicited several self-regulatory behaviors involving the brain, body, and environment. Regarding her genre environment, she borrowed one genre she knows, the list on a continuum, to help her write another she is struggling with, the literature review. Regarding self-regulation, she physically arranged (body, behavior) her office (physical environment) to manage the social action of the sub-genre. In doing so, she managed her attention, her emotions (“to motivate me”), and her embodied thinking (the stacks of articles and the figure). Interestingly, she did not report a felt sense of thinking or writing but only of physically arranging objects in her immediate environment, drawing brackets on a post-it, and the positive emotional valence that accompanied the renewed motivation.

In phenomenological terms, Kel experienced motivation as plural, complex, even competing “motivational states” (Deci and Ryan, 1981), shifting from moment to moment, intention to intention, during the process of writing. At various timescales, from a single slow-down/restart to a whole dissertation writing process, various interweaving intrinsic and extrinsic motivations may take the fore at any moment and, with them, various identities and various senses of motivation (or its lack)—motivational states. Kel describes this often in terms of competing pulls of teaching, research, family, and personal care impinging on her attention, on her felt sense, moment to moment. However, the motivational states are, for the writer, anchored in the stabilized-for-now object of her activity, the genre as social action, and the subgenres that exist in the writer’s genre system and broader BBE system of motivation. In this sense, genre as social action can be seen as a key to motivation.

## 4 Discussion

The WWC model attempts to merge sociocultural and cognitive perspectives. This article attempts to elaborate further implications of it using genre theory and SDT by explicating the motivational processes of writing within communities. This discussion suggests implications of IP cognitive and SDT research for empirical sociocultural research and implications of genre as social action for IP cognitive studies research, along with suggestions for pursuing shared areas of interest between genre theory and SDT.

### 4.1 Implications for sociocultural studies

Socio-cultural studies of motivation have been overwhelmingly qualitative, which limits the generalizability of the findings (Haswell, 2005; Yin, 2014). IP cognitive and SDT research can add a quantitative element to socio-cultural approaches to increase the power and reach of the sociocultural approach (e.g., MacArthur et al., 2016). Graham and others have begun that work already. For example, to test socio-cultural claims about the effects of macro-level features on meso-level classroom practices, Hsiang and Graham (2016) surveyed teachers to see if particular government and educational policy features influenced how writing was taught and varied across locations.

The dominant mainline socio-cultural approaches to motivation, from Britton (1975), have tended to view classroom genres and the social actions or practices they embody (e.g., assessment essays, reports demonstrating knowledge) as generally demotivating. Moreover, many sociocultural pedagogical innovations are an attempt to motivate students by having them write personal or “real-world” genres, often in situations where the task is collaborative or the topic is chosen by the student (or presented in a way to spark interest) (Hidi and Boscolo, 2006).

In this view, how students enactively perceive genres differently from teachers and other students becomes a crucial driver of how they write (or do not) them. Current research increasingly finds that motivation is “dynamic, context-sensitive, and changeable” (Maclellan, 2005, p. 194). If motivation is about making meanings through social action, then the varying ways students perceive genres with their social actions can be crucial. One use of phenomenology and genre as social action is to complicate the concepts of personal and real-world genres and the school-based genres they are compared unfavorably with in dominant socio-cultural approaches. Two sociocultural studies will illustrate this.

The phenomenological view allows researchers and teachers to deepen their understanding of classroom genres and motivation. Genres as social actions may align—and skew—the motives of the teacher and student. For example, Yañez and Russell (2009) studied a journalism major taking an Irish history course (as a general education elective) who was highly motivated to write a paper on it because she saw it as preparation for a career where journalistic standards would require her to tell a story “objectively.” However, the teacher was motivated by professional standards of academic history that assumed there was no objective truth but only different versions that needed to be accounted for. Different disciplinary perspectives produced profoundly different perceptions of the same classroom assignment. If teachers realize the ways students are appropriating their classroom genres, they can change the genres or reframe them for students to increase their motivation.

Information-processing cognitive and SDT research can provide a way to operationalize and quantify for research purposes the perceptions and effects of genre as social action on motivation. More extensive studies might survey students to see how they perceive the genre(s) as social action they intend(ed) to write, for example, to get at their varying motivational states and contradictions within them, which, as WWC points out, are often in play.

Similarly, in Gere et al.’s (2018) study, “A Tale of Two Prompts,” students in a university statistics course were asked to show their understanding of statistics concepts by writing (1) an email to grandparents analyzing studies of the effects of caffeine and recommending when and how much coffee to drink and then (2) a memo to a Tour de France team analyzing studies of the effects of dark chocolate on athletic performance with diet recommendations for the team. Students did far better on the second task because, the authors argued, the second task created “a clear through-line from present work to future work,” while “the grandparent assignment was a kind of cul-de-sac; a worthy end, perhaps, but an end in itself and not a means of writing their way into a professional world” (pp. 164–165). The students could not “see themselves” explaining statistical concepts to their grandparents about chocolate, but they could see it for a professional team making a high-stakes decision on nutrition.

The authors of the study mentioned motivation explicitly only twice; instead, they described the difference in “aspirational function” or “anticipatory socialization for career mobility” (Gere et al., 2018, p. 164). How the students perceive the genre as social action in relation to their future identity is crucial. The authors conclude, “A useful question to ask about an assignment is what kind of aspirational quality it has and how it might be perceived as a scaffold to a desirable future role. How an assignment is constructed can go a long way toward supporting students in making meaning of their learning and conveying their knowledge of course concepts” (Gere et al., 2018, p. 165). Taking into account the motivational aspects of genre as social action may clarify such aspects of classroom genres and tease out the ways they are demotivating (for some) and might be made more motivating by changing or reframing them. That reframing might grow out of the differences in students’ perception of the genre as social action of an email to grandparents and a memo to an organization—the latter wielding much more power and thus consequences.

These and other socio-cultural qualitative studies might benefit from or inspire quantitative studies in the SDT tradition as they are very much about intrinsic and extrinsic motivation. Ryan and Deci’s (2004) analysis of the extrinsic/intrinsic continuum might help describe the motivational differences in the assignment genres—and perhaps offer a more precise framework for generating testable hypotheses for future quantitative research to delineate the ways active genre perception affects and is affected by motivation.

Self-Determination Theory and research methods could partially overcome the chief limitation of qualitative research usually employed to study genre as social action—the lack of generalizability. Researchers have developed over 30 questionnaire scales to measure SDT constructs, several of which would be useful in larger-scale studies of motivation and genre (Metrics and Methods, n.d.).

Quantitative empirical research on motivation in the tradition of SDT shares much with socio-cultural research regarding assumptions about the roles of agency and autonomy in motivation and the dynamic interplay of brain, body, and environment (context) in meaning-making and self-regulation. It might readily inform socio-cultural studies of L1 writing, as it has yet to do thus far, apart from a few exceptions (Robinson, 2009; DeCheck, 2012; Kirchhoff, 2016; Williams, 2018; Feigenbaum, 2021).

Because SDT and genre as social action/dialogic share basic assumptions, it is worth noting again that the continental dialogic tradition of phenomenology, represented most relevantly here, perhaps, by Linell (2009), has significant similarities with the North American tradition of genre as social action and might also benefit from SDT research for the same reasons. Furthermore, the many second language acquisition studies using SDT might provide models for L1 writing studies.

### 4.2 Implications for IP cognitive studies

Similarly, IP cognitive and SDT researchers might benefit from a phenomenological analysis that explicates the relationship between students’ perception of the genre as social action and their construal of—and motivations for—pedagogical interventions or experimental tasks.

For example, in one of the only studies of student L1 writing motivation using SDT, Graham and colleagues took up an important issue in both socio-cultural and IP cognitive approaches to motivation: the effects of choice and preference in an argumentative writing task on student motivation and performance (Aitken et al., 2022; Aitken and Graham, 2023). Some 224 US undergraduate students in an introductory course on special education participated. In each of the two 75-min classes, students were given a case study on a controversial issue raised in the course material (ADHD medication for a second grader and a more restrictive environment for a student with behavioral issues). In the first 60 min, a “guest lecturer” (the first author) introduced the case, and students discussed it with other students who took differing positions and made notes for writing. They then wrote a 25-min essay arguing for a position on each of the two topics. Students were divided into two groups. For the first case study, one group was assigned a position, and the other chose a position. For the second case study, the groups were reversed. Before the class sessions, students completed measures of writing self-efficacy and knowledge of the two topics, and afterward, their essays were scored holistically.

The quantitative analysis (Aitken et al., 2022) found that the effects of choice on writing performance were limited, while the qualitative analysis (Aitken and Graham, 2023), using SDT extensively, provided important insights into the effects of choice and preference on motivation. Researchers predicted that “choice would have a statistically significant impact on writing quality because, following self-determination theory, an autonomy-enhancing technique, such as choice, should enhance students’ intrinsic motivation for the task to be completed” (Aitken et al., 2022, p. 1856). However, they “did not find a main effect for choice, drawing into question the common contention among many writing experts and teachers that choice is a universally effective tool for improving writing” (Aitken et al., 2022, p. 1856). I suggest that analyzing the students’ perception of the genre as social action might offer further insights into the study results and its use of SDT. How did the students perceive the genre as social action, and with what effects on their motivational beliefs?

The researchers rightly point out an advantage of their study over previous studies: it “was conducted in a real classroom context rather than as a contrived assessment to test students’ writing competence” (Aitken et al., 2022, p. 1857). Despite this clear and potentially significant advantage in context authenticity, the students’ perception of the task environment—the genre as social action—may have blunted (mediated) the effect of choice—because it reduced their autonomy. Students in the “choice” groups were not choosing a topic but only a position on an assigned topic. In the qualitative study, students praised opportunities to choose a topic. However, several did not, with one student who did not “see choosing her position on an assigned topic as a “real” choice even though she recognized that it was technically a choice; maybe, just not a meaningful choice,” and several expressed dislike at being forced to write on a topic as it reduced their autonomy (Aitken and Graham, 2023, p. 311).

Moreover, students in the “choice” groups were not choosing a genre but were assigned one, further reducing choice. The students were actually given two genres, a pretend one masking a real one—an ambiguity common in classroom genres. The prompts were presented as letters: “Pretend you are Mr. Lars and write a letter to (your wife) by arguing that you should (should not) put your son on medication for his ADHD symptoms” (Aitken et al., 2022, p. 1849). However, the prompt refers to the task as a “persuasive essay” or “argumentative essay.” Furthermore, writing a letter to one’s spouse about such an important and emotionally charged decision about a child—rather than face-to-face or phone communication—puts this in the phenomenal world of the classroom exercise, not the family, where the stakes and emotional valences are different. Indeed, whom are the students persuading, and of what? Students may have felt—with reason—that there is no expected meaningful communication outside classroom/experimental meanings of content learning activities or writing exercises (Magnifico, 2010).

Moreover, no grades were attached, and the students were told their essays would not be read by their partners, so even the motive of persuading the teacher and peers is not live but imaginary. The methods presented in the studies do not specify any purpose the students were given in the instructions/framing of the intervention. Logically, the students might have inferred that the goal of this task—the genre as social action—is to learn about the issues involved in the syllabus topics, not to communicate or write well. If that is the case, some students may have been motivated to try harder on the position they disagreed with. As the authors point out (Aitken et al., 2022, p. 1858), the qualitative study supports this view as a significant number of the interviewees saw “experiencing new perspectives” as a benefit and so tried to “knock it out of the park”—a logical consequence of perceiving the genre as social action as a discussion- or writing-to-learn exercise. Though the authors do not mention SDT in their analysis of the benefits of no choice, it is possible to see how these students were meeting their need for autonomy by choosing to find the benefits, making the genre not a meaningless classroom exercise but an opportunity or choice to exercise their writing powers. Thus, “autonomy-enhancing techniques” “for optimal writing outcomes” might include not only the two analyzed in the study—choice of topic and choice of position—but also a choice of genre as social action—how the students and the teacher/researcher choose to frame or reframe the action phenomenologically.

Similarly, in a study of Portuguese students in grades 5 through 8 who wrote one narrative text (“Tell a story about a child who found a wounded animal”) and one opinion text (“What is your opinion about children practicing sport every day?”), Camacho et al. (2022) hypothesized that the students’ implicit theories would be significantly associated with performance-oriented goals. This hypothesis was not supported. That is, students whose score on the implicit theories measure indicated they believed their writing skills tend to be fixed (rather than malleable and thus incrementally improvable) also tended to score lower on the goals measure that indicated their intentions or goals when they write are to perform better than other students (performance-approach goals)—a finding that contradicted earlier research in other subject areas such as math. Moreover, the study “indicated a direct, negative relation between performance-approach goals and narrative text quality” (and a negative though less significant relation on opinion text quality) (Camacho et al., 2022, p. 9).

Importantly, however, Camacho et al. (2022, p. 5) “used a writing performance measure which was only scored for research purposes and had no influence on students’ grades.” As the authors point out, the lack of a grade “may partially explain the non-significant relations between performance-based goals (either approach or avoidance) and writing performance” (Camacho et al., 2022, p. 8). The authors point out that another similar study, with older students, “used a graded writing assignment with influence for grades” and found an association between performance-approach goals and text quality (Camacho et al., 2022, p. 9).

In phenomenological terms, the genre as social action that students perceived may not have been either opinion or narrative about either wounded animals or sports participation; it may have been perceived as doing a classroom exercise for the researchers. Students with mastery goals seem to have perceived the social action as practicing writing skills. In contrast, the students with performance-based goals seem to have perceived the social action as low-value “busy work” unrelated to their grades. As Camacho et al. (2022, p. 2) point out, “Implicit theories can be domain-specific as one student may believe that ability in one school domain is malleable (e.g., writing), while ability in another domain is innate.” A phenomenological analysis suggests that implicit theories may also vary across genres as social actions. If so, this might be an important variable or complex of variables for generating new testable hypotheses or reanalyzing existing data.

Viewing a task (environment) as a genre as social action might further elaborate models of the components of competence that affect motivation beyond the trichotomous model developed by Elliot et al. (2011), which Camacho et al. (2022) used. Elliot et al. (2011) developed a 3 × 2 goal model, “which is ingrained in the definition (task, self, or other) and valence (positive or negative) components of competence, encompassing six goals (i.e., task-approach, task-avoidance, self-approach, self-avoidance, other-approach, and other-avoidance).” A phenomenological analysis might propose other definitions and valences by elaborating on the concept of a task as a genre as social action. For example, the task might be viewed differently from a proximal or distal perspective and its motivations as different in valence and degree, accordingly. A trivial and negative task from the immediate perspective of the classroom might be highly salient and positive from the perspective of another social action, such as an aspirational socialization perspective. Similarly, the self of the research subject, the self of the student seeking a good mark, and the aspirational self of the imagined future professional might elicit different motivations. Finally, performing for others might mean performing for teachers, classmates, distal readers (such as Kel’s article authors), or imagined future colleagues (Kel’s imagined collaborators).

One implication of beginning with an analysis of participants’ (students, teachers, researchers, etc.) perception(s) of the genre as social action is that the genre and its framing (actual and fictional) become a part of the design of classroom activities and research studies. For example, Wardle analyzes what she calls “mutt genres”: “genres that share superficial conventions with other genres” but have been stripped of their original social action. Mutt genres “mimic genres that mediate activities in other activity systems, but within the (new) activity system their purposes and audiences are vague or even contradictory. They are quite different from and serve very different purposes in (writing classrooms or research) than they do in other disciplinary activity systems” (Wardle, 2009, p. 774).^8^ Research and pedagogy in both socio-cultural and IP cognitive approaches might use phenomenological genre analysis to find potential pitfalls in assignments and their framing to prevent—or retrospectively diagnose—confusions and contradictions that impede writing development or research progress and to generate testable hypotheses on the effects of student perception of the task and task environment, framed as genre as social action.

### 4.3 Shared areas of interest between the SDT and genre as social action

The deep shared roots of genre as social action and SDT in existential phenomenology suggest fertile ground for research into writing and cognition—but embodied, embedded cognition in the tradition of Luria (Bazerman, 2013; Portanova et al., 2018).

One shared area to explore is mindfulness in research and pedagogical interventions. Since Maturana and Varela’s (1991) work in the early 1990s, the phenomenological tradition has been occupied with mindfulness, including relationships with Eastern traditions. Two decades before that, the therapist Gendlin (1982) developed a mindfulness technique called Focusing out of the phenomenological concept of the felt sense, which he further developed as a technique specifically to help writers, Thinking at the Edge (2004). Gendlin’s (2004) student, the pioneering writing researcher Sondra Perl, developed the Felt Sense exercises for use in writing classrooms. Ryan and Deci (2004) refer approvingly to Maturana and Varela (1991) and to Gendlin (1982) when they compare SDT to existential/phenomenological approaches to mindfulness. This is not surprising as both SDT and felt-sense approaches recommend that people become mindful when deciding when something is authentic. Both attend not only to the brain but also to what resonates in the whole body and beyond, the phenomenal self, orchestrating the body, brain, and environment. One area to be explored is mindfulness as a self-regulating strategy to be developed in classrooms and other pedagogical settings, as Perl (2004) does. Self-regulated strategy development (SRSD) research (Ennis et al., 2014) notes that much of the self-regulation and self-determination language of the SRSD model, including the use of positive self-statements and self-questioning (Graham and Harris, 1996), mirrors language used in mindfulness training. One essential addition of mindfulness training is its emphasis on becoming aware of the body and emotions, explicitly locating and harnessing the felt sense of writing some genre, as in Horwitz et al.’s (2018) intervention study of writer’s block.

Another area where research might overlap is in the neuro-substrate of motivation. SDT has attempted to “map the phenomenology of intrinsic motivation onto the neural substrates of motivational processes that are encompassed by intrinsic motivation” (Di Domenico and Ryan, 2017, p. 2). Although no neuro-imaging studies (to my knowledge) have specifically studied writing motivation directly, studies that use neuroimaging to track brain activity as subjects carry out tasks that suggest analogs to intrinsic motivation have produced exciting results. They suggest that well-documented neural processes such as the SEEKING and dopamine systems are at work. “A complementary approach to theorizing about the neural systems that support intrinsic motivation is to map its phenomenology with the activity of large-scale neural networks” (Di Domenico and Ryan, 2017, p. 9). For example, researchers have mapped the “neural correlates of intrinsic motivation by comparing patterns of neural activity when undergraduate students imagined themselves performing intrinsically motivating writing activities (e.g., “writing an enjoyable article”) and extrinsically motivating writing activities (e.g., “writing an extra-credit article”). Most prominently, these studies found preferential activity within insular regions when participants imagined the enactment of intrinsically motivating activities” (p. 9).

The phenomenological tradition has for 20 years pursued the third-person neural correlates of first-person and second-person descriptions of experience. Researchers use phenomenological descriptions—the description of one’s own mental phenomena “bracketed off” from immediate action or interviews to elicit such descriptions—in conjunction with neural imaging to produce “neuro-phenomenology,” a term coined in the mid-1990s by the Chilean cognitive neuroscientist Varela (1996). The goal of neurophenomenology is to use first-person phenomenological description (or second-person interviews) to expand and enrich third-person accounts drawn from the experimental methods of neuroscience and vice versa (Gallagher, 2012, pp. 36–37, 107–108).

Much neuro-phenomenology research studies meditation and other mindfulness practices. In the classic study of Nepalese monks (Thompson, 2007), neuroscientists noted that the monks claimed their meditation enhanced their mental “clarity.” To investigate this further, the neuroscientists measured the monks’ brain activity through electrodes while asking them to rate their clarity feelings on a Likert scale before, during, and after meditation. The self-reported subjective clarity ratings of experienced monks corresponded with an increase in high-amplitude gamma synchrony, which was not observed in novice monks, who served as the control group. The study’s author emphasizes the importance of the first-person phenomenological descriptions in understanding the changes in brain activity as these subjective reports demonstrate that these changes are indeed happening, which would be unclear (noise in the data) to neuroscientists using only third-person methods. Similar neurophenomenological studies have been conducted in various fields, particularly pain management. There have been no studies of the neural substrates of writing thus far. However, there have been studies of writing using phenomenological description in conjunction with other third-person methods, such as eye tracking, keystroke logging, and video (Gallagher et al., 2015; Horwitz et al., 2018), to study surveillance anxiety and writer’s block.

Neuroscientific studies of both mindfulness and pre-reflective awareness concerning writing might well provide insights with explanatory power for both phenomenological and social cognitive theories. Important work on the SEEKING system, for example, undergirds theorizing on the role of emotion in motivation within research on both the neural substrates of SDT in the IP cognitive tradition and ecological and radical embodied psychology in the phenomenological and socio-cultural traditions (Gabriel, 2021) (see Portanova et al., 2018 on cognition in writing studies and Clark, 2022 on writing and neuroscience research).

## 5 Conclusion

Students’ perception (or, often, varying perceptions) of the genre as social action may profoundly affect their motivation and thus, potentially, their growth. Taking genre as social action as a construct for writing research can add to attempts to bridge socio-cultural and IP cognitive traditions and allow each to deepen their insights in terms of theory, research, and pedagogical (re)design.

## Data availability statement

The original contributions presented in this study are included in this article/supplementary material, further inquiries can be directed to the corresponding author.

## Ethics statement

The studies involving humans were approved by the Iowa State University Office of Research Ethics. The studies were conducted in accordance with the local legislation and institutional requirements. The participants provided their written informed consent to participate in this study. Written informed consent was obtained from the individual(s) for the publication of any potentially identifiable images or data included in this article.

## Author contributions

The author confirms being the sole contributor of this work and has approved it for publication.

## Footnotes

1 This article focuses on the North American socio-cultural research on genre (Bazerman, 1994, 2013; Russell, 1997). A broader continental tradition of dialogic semiotic theory and research on language in use takes what are in some ways similar approaches to genre (Berge, 1988, 1993; Linell, 2009). Both have roots in phenomenology and the cultural psychology of Vygotsky, and both have been profoundly influenced by the Bakhtin Circle’s work. Beyond that, many phenomenological perspectives exist on these linguistic and semiotic issues, from Jakobson’s phenomenological structuralism (Holenstein, 1976) to French *genres de texte* (Bota and Bronckart, 2007). Because the differences are significant, I cannot, in this space, do much more than acknowledge this broader scope. However, I will refer to continental theory and research that particularly resonate with the North American tradition.

2 Other important theoretical attempts to bridge IP cognitive and sociocultural approaches to writing include Bazerman (2013); Portanova et al. (2018); Klein (2019); Mitchell et al. (2019) and, notably, a special issue of Educational Psychologist (Turner and Nolen (2015)).

3 By phenomenology, I mean a method of doing both philosophy and empirical inquiry that describes “how things appear, show, or give themselves in lived experience or in consciousness” (van Manen, 2017, p. 775), a first-person (or second-person interview-based) description of the “felt sense” of some phenomenon, some particular experience. The goal is in-depth understanding of and meaningful insight into some aspect of the experience that cannot be fully understood from an external, third-person perspective. There have been many North American empirical studies of the phenomenology of writing: handwriting versus typewriting (Chandler, 1992; Haas, 1996), perception of errors in writing (Williams, 2011), freewriting (Elbow, 1989), genre and transfer across the lifespan (Dippre, 2019), motivation in problem-solving (Williams, 2011), qualitative methodology (Prior, 2014), and digital writing (van Manen and Adams, 2009). Moreover, continental theoretical literature on the phenomenology of writing is vast, e.g., Derrida’s (2001) *Writing and Difference*.

4 Homeostasis has figured in previous theories of writing processes (Nystrand, 1989) and of rhetorical exigence (e.g., Hunsaker and Smith, 1976; Oakley, 1999), though in different ways than presented here.

5 Elsewhere, I (Russell, 2019) have explored the relation between genre as social action and Hayes’s (2012) three-level IP cognitive model of writing processes, which forms the basis of the WWC analysis of individual writing processes. The top level, what Hayes calls the control level, involves 1. motivation, 2. goal setting (plan write revise), 3. current plan, and 4. writing schemas. These all, I argued, can be seen from a phenomenological point of view as aspects of active genre perception—genreing. They can be understood not only by IP cognitive load theory but also by evolutionary cognitive load theory, in that Hayes’s control level utilizes functional systems that evolved before writing, ontogenetically and phylogenetically, to manage the cognitive load, such as typifying perception, problem-solving action, sociality, cooperation, indexical pointing, and, of course, languaging. Writers responding to their perception of a genre as social action can also be seen as motivating and managing the other elements of the Hayes model: writing processes (Hayes’ second level, which includes task environment) and the writer’s resources (Hayes’ third level, which includes working memory, long-term memory, reading, and attention), similar to the WWC model.

6 There are numerous other ways of categorizing pragmatic discourse that have been used to structure curriculum and assessment, such as the Norm project. See Berge et al. (2019) and particularly Berge et al. (2016), which reviews previous efforts before proposing a new model of theorizing writing.

7 Functional systems are analyzed by Vygotsky, his neuroscientist collaborator Luria, and his social systems collaborator Leontiev (Bazerman, 2013).

8 Berge et al. (2016) make a similar point (p. 14), though they argue for a model that goes beyond genre, to categorize and simplify the acts and purposes for writing in a way that facilitates writing pedagogy and assessment (Berge et al., 2019).

## References

[B1] Aitken A. Graham S. (2023). Perceptions of choice in writing of university students. *J. Writ. Res.* 15 225–332. 10.17239/jowr-2023.15.02.03

[B2] Aitken A. A. (2023). “More motivating than cherry pie? The Writer(s) within community model of writing through a motivation theory lens,” in *The hitchhiker’s guide to writing research*, eds Liu X. Hebert M. Alves R. A. (Berlin: Springer).

[B3] Aitken A. A. Graham S. McNeish D. (2022). The effects of choice versus preference on writing and the mediating role of perceived competence. *J. Educ. Psychol.* 114 1844–1865. 10.1037/edu0000765

[B4] Bandura A. (1962). “Social learning through imitation,” in *Nebraska symposium on motivation*, ed Jones M. R. (Lincoln: University of Nebraska Press), 211–274.

[B5] Bawarshi A. S. (2003). *Genre and the invention of the writer: Reconsidering the place of invention in composition.* Logan, UT: Utah State University Press.

[B6] Bazerman C. (1988). *Shaping written knowledge.* Madison, WI: University of Wisconsin press.

[B7] Bazerman C. (1994). “Systems of genres and the enactment of social intentions,” in *Genre and the new rhetoric*, eds Freedman A. Medway P. (Milton Park: Taylor & Francis), 79–101.

[B8] Bazerman C. (2013). *A theory of literate action: Literate action volume 2.* Anderson, SC: Parlor Press LLC.

[B9] Berge K. L. (1988). *Skolestilen som genre [The school essay as genre].* Oslo: Cappelen.

[B10] Berge K. L. (1993). “The diachrony of textual norms; or why do genres change?,” in *Changing English*, ed. Graddol D. (Routledge: London), 217–228.

[B11] Berge K. L. Evensen L. S. Thygesen R. (2016). The wheel of writing: A model of the writing domain for the teaching and assessing of writing as a key competency. *Curric. J.* 27 172–189.

[B12] Berge K. L. Skar G. B. Matre S. Solheim R. Evensen L. S. Otnes H. (2019). Introducing teachers to new semiotic tools for writing instruction and writing assessment: Consequences for students’ writing proficiency. *Assess. Educ.* 26 6–25. 10.1080/0969594X.2017.1330251

[B13] Bitzer L. F. (1968). The rhetorical situation. *Philos. Rhetor.* 1 1–14.

[B14] Bota C. Bronckart J.-P. (2007). Volochinov et bakhtine: Deux approches radicalement opposées des genres de ıtextes et de leur statut. *Revue des Linguistes de l’université Paris X Nanterre* 56 73–89. 10.4000/linx.360

[B15] Britton J. (1975). *The Development of writing abilities (11–18).* Milton Park: Taylor & Francis.

[B16] Camacho A. Alves R. A. Daniel J. R. De Smedt F. Van Keer H. (2022). Structural relations among implicit theories, achievement goals, and performance in writing. *Learn. Individ. Differ.* 100:102223. 10.1016/j.lindif.2022.102223

[B17] Chandler D. (1992). The phenomenology of writing by hand. *Digit. Creat.* 3 65–74.

[B18] Clark I. L. (2022). *Writing, imitation, and performance: Insights from neuroscience research.* Milton Park: Taylor & Francis.

[B19] DeCheck N. (2012). The power of common interest for motivating writers: A case study. *Writ. Center J.* 32 4. 10.7771/2832-9414.1852 29356800

[B20] Deci E. L. Ryan R. M. (1981). *Curiosity and self-directed learning: The role of motivation in education.* Washington, DC: ERIC Publications.

[B21] Deci E. L. Ryan R. M. (2013). *Intrinsic motivation and self-determination in human behavior.* Berlin: Springer Science & Business Media.

[B22] Derrida J. (2001). *Writing and difference.* London: Routledge.

[B23] Di Domenico S. I. Ryan R. M. (2017). The emerging neuroscience of intrinsic motivation: A new frontier in self-determination research. *Front. Hum. Neurosci.* 11:145. 10.3389/fnhum.2017.00145 28392765 PMC5364176

[B24] Dippre R. J. (2019). *Talk, tools, and texts: A logic-in-use for studying lifespan literate action development.* Louisville, KY: WAC Clearinghouse.

[B25] Dreyfus H. L. (2002). Intelligence without representation – Merleau-Ponty’s critique of mental representation. The relevance of phenomenology to scientific explanation. *Phenomenol. Cogn. Sci.* 1 367–383. 10.1023/A:1021351606209

[B26] Dryer D. B. Russell D. R. (2018). “Attending to phenomenology: Rethinking cognition and reflection in North American Writing Studies,” in *Contemporary perspectives on cognition and writing*, eds Portanova P. Rifenburg J. M. Roen D. H. (Louisville, KY: WAC Clearinghouse), 57–76.

[B27] Elbow P. (1989). Toward a phenomenology of freewriting. *J. Basic Writ.* 8 42–71.

[B28] Elliot A. J. Murayama K. Pekrun R. (2011). A 3 × 2 achievement goal model. *J. Educ. Psychol.* 103 632–648.

[B29] Ennis R. P. Harris K. R. Lane K. L. Mason L. H. (2014). Lessons learned from implementing self-regulated strategy development with students with emotional and behavioral disorders in alternative educational settings. *Behav. Disord.* 40 68–77.

[B30] Feigenbaum P. (2021). Welcome to “Failure Club”: Supporting intrinsic motivation, sort of, in college writing. *Pedagogy* 21 403–426.

[B31] Gabriel R. (2021). The motivational role of affect in an ecological model. *Theory Psychol.* 31 552–572.

[B32] Gallagher S. (2012). *Phenomenology.* London: Palgrave Macmillan.

[B33] Gallagher S. Janz B. Reinerman L. Trempler J. Bockelman P. (2015). *A neurophenomenology of awe and wonder: Towards a non-reductionist cognitive science.* Berlin: Springer.

[B34] Gendlin E. T. (1982). *Focusing.* New York, NY: Bantam.

[B35] Gendlin E. T. (2004). Introduction to thinking at the edge. *Folio* 19 1–8.

[B36] Gere A. R. Knutson A. V. Limlamai N. McCarty R. Wilson E. (2018). A tale of two prompts: New perspectives on writing-to-learn assignments. *WAC J.* 29 147–188. 10.37514/WAC-J.2018.29.1.07

[B37] Graham S. (2018). A revised writer (s)-within-community model of writing. *Educ. Psychol.* 53 258–279.

[B38] Graham S. Harris K. R. (1996). “Self-regulation and strategy instruction for students who find writing and learning challenging,” in *The science of writing: Theories, methods, individual differences, and applications*, eds Levy C. M. Ransdell S. (Mahwah, NJ: Lawrence Erlbaum Associates, Inc), 347–360.

[B39] Greeno J. G. Engeström Y. (2014). “Learning in Activity,” in *The Cambridge Handbook of the Learning Sciences*, 2nd Edn, ed. Sawyer R. K. (Cambridge, MA: Cambridge University Press), 128–148. 10.1017/CBO9781139519526.009

[B40] Gregersen A. (2011). Genre, technology and embodied interaction: The evolution of digital game genres and motion gaming. *J. Med. Commun. Res.* 27 94–109.

[B41] Haas C. (1996). *Writing technology: Studies on the materiality of literacy.* London: Routledge.

[B42] Hasson U. Egidi G. Marelli M. Willems R. M. (2018). Grounding the neurobiology of language in first principles: The necessity of non-language-centric explanations for language comprehension. *Cognition* 180 135–157. 10.1016/j.cognition.2018.06.018 30053570 PMC6145924

[B43] Haswell R. H. (2005). NCTE/CCCC’s recent war on scholarship. *Writ. Commun.* 22 198–223. 10.1177/0741088305275367

[B44] Hayes J. R. (2012). Modeling and remodeling writing. *Writ. Commun.* 29 369–388. 10.1177/0741088312451260

[B45] Hidi S. Boscolo P. (2006). *Writing and motivation.* Leiden: Brill.

[B46] Holenstein E. (1976). *Roman Jakobson’s approach to language: Phenomenological structuralism.* Bloomington, IN: Indiana UP.

[B47] Horwitz E. B. Stenfors C. Osika W. (2018). Writer’s block revisited: A micro-phenomenological case study on the blocking influence of an internalized voice. *J. Conscious. Stud.* 25 9–28.

[B48] Hsiang T. P. Graham S. (2016). Teaching writing in grades 4–6 in urban schools in the greater China region. *Read. Writ.* 29 869–902.

[B49] Hunsaker D. M. Smith C. R. (1976). The nature of issues: A constructive approach to situational rhetoric. *Western J. Commun.* 40 144–156.

[B50] Hyland K. (2000). *Disciplinary discourses: Social interactions in academic writing.* Harlow: Longman.

[B51] Kirchhoff L. (2016). Motivation in the writing centre: A peer tutor’s experience. *J. Acad. Writ.* 6 31–40.

[B52] Klein P. D. (2019). “Integrating social and psychological perspectives on writing as a learning activity,” in *Knowing writing: Writing research across borders*, eds Bazerman C. Carlino P. Castello M. Narvez E. Tapia-Ladino M. Pinzon B. Y. G. (Louisville, KY: The WAC Clearinghouse), 393–415. 10.37514/INT-B.2019.0421.2.19

[B53] Lee S. W. (2019). A copernican approach to brain advancement: The paradigm of Allostatic orchestration. *Front. Hum. Neurosci.* 13:129. 10.3389/fnhum.2019.00129 31105539 PMC6499026

[B54] Lieberman P. (2007). The Evolution of human speech: Its anatomical and neural bases. *Curr. Anthropol.* 48 39–66. 10.1086/509092

[B55] Linell P. (2009). *Rethinking language, mind, and world dialogically.* Charlotte, NC: IAP.

[B56] MacArthur C. A. Philippakos Z. A. Graham S. (2016). A multicomponent measure of writing motivation with basic college writers. *Learn. Disabil. Q.* 39 31–43.

[B57] Maclellan E. (2005). Academic achievement: The role of praise in motivating students. *Act. Learn. Higher Educ.* 6 194–206. 10.1177/1469787405057750

[B58] Magnifico A. M. (2010). Writing for whom? Cognition, motivation, and a writer’s audience. *Educ. Psychol.* 45 167–184.

[B59] Maturana H. R. Varela F. J. (1991). *Autopoiesis and cognition: The realization of the living*, Vol. 42. Berlin: Springer Science & Business Media.

[B60] Mehlenbacher A. R. (2019). *Science communication online: Engaging experts and publics on the internet.* Columbus, OH: The Ohio State University Press.

[B61] Merleau-Ponty M. (1964). *The primacy of perception: And other essays on phenomenological psychology, the philosophy of art, history, and politics.* Evanston, IL: Northwestern University Press.

[B62] Merleau-Ponty M. (2013). *Phenomenology of perception.* London: Routledge.

[B63] Merton R. K. (1968). *Social theory and social structure.* New York, NY: Free Press.

[B64] Metrics and Methods (n.d.). *Questionnaires – selfdeterminationtheory.org.* Available online at: https://selfdeterminationtheory.org/questionnaires/ (accessed October 27, 2023).

[B65] Miller C. R. (1984). Genre as social action. *Q. J. Speech* 70 151–167.

[B66] Mitchell K. M. McMillan D. E. Lobchuk M. M. (2019). Applying the ‘Social Turn’ in writing scholarship to perspectives on writing self-efficacy. *J. Learn. Dev. Higher Educ.* 15:15. 10.47408/jldhe.v0i15.512

[B67] Nagataki S. Hirose S. (2007). Phenomenology and the third generation of cognitive science: Towards a cognitive phenomenology of the body. *Hum. Stud.* 30 219–232.

[B68] Nystrand M. (1989). A social-interactive model of writing. *Writ. Commun.* 6 66–85. 10.1177/0741088389006001005

[B69] Oakley T. V. (1999). The human rhetorical potential. *Writ. Commun.* 16 93–128. 10.1177/0741088399016001005

[B70] Paré A. (2014). Rhetorical genre theory and academic literacy. *J. Acad. Lang. Learn.* 8 A83–A94.

[B71] Paré, A. Starke-Meyerring D. McAlpine L. (2007). The dissertation as multi-genre: Many readers, many readings. *Perspect. Writ.* 179–193.

[B72] Perl S. (2004). *Felt sense: Writing with the body.* Portsmouth, NH: Boynton/Cook Heinemann Portsmouth.

[B73] Portanova P. Rifenburg J. M. Roen D. H. (2018). *Contemporary perspectives on cognition and writing.* Louisville, KY: WAC Clearinghouse.

[B74] Prior P. (2009). “From speech genres to mediated multimodal genre systems: Bakhtin, Voloshinov, and the question of writing,” in *Genre in a changing world*, eds Bazerman C. Bonini A. Figueiredo D. (Louisville, KY: WAC Clearinghouse), 17–34.

[B75] Prior P. (2014). “Combining phenomenological and sociohistoric frameworks for studying literate practices: Some implications of deborah brandt’s methodological Trajectory,” in *Literacy, economy, and power: Writing and research after “literacy in american lives*, eds Duffy J. Christoph J. N. Goldblatt E. Graff N. Nowacek R. S. Trabold B. (Carbondale, IL: Southern Illinois University), 166–182.

[B76] Quote Investigator^®^ (2011). *I Don’t want to belong to any club that will accept me as a member.* https://quoteinvestigator.com/2011/04/18/groucho-resigns/ (accessed 2011, April 18, 2011).

[B77] Robinson H. M. (2009). Writing center philosophy and the end of basic writing: Motivation at the site of remediation and discovery. *J. Basic Writ.* 28 70–92.

[B78] Russell D. R. (1997). Rethinking genre in school and society: An activity theory analysis. *Writ. Commun.* 14 504–554.

[B79] Russell D. R. (2019). “Perception and recognition of textual genres: A phenomenological approach,” in *Knowing writing: Writing research across borders*, eds Bazerman C. Yaneth B. Pinzón G. (Denver, CO: Colorado State University Press), 163–198. 10.37514/INT-B.2019.0421.2.20

[B80] Ryan R. M. (2017). *Self-determination theory: Basic psychological needs in motivation, development, and wellness.* New York, NY: Guilford Press.

[B81] Ryan R. M. Deci E. L. (2004). “Autonomy is no illusion: Self-determination theory and the empirical study of authenticity, awareness, and will,” in *Handbook of experimental existential psychology*, eds Koole S. L. Greenberg J. Pyszczynski T. A. (New York, NY: Guilford Press), 449–479.

[B82] Schütz A. (1951). Making music together: A study in social relationship. *Soc. Res.* 18 76–97.

[B83] Schutz A. (1973). *The structures of the life-world.* Evanston, IL: Northwestern University Press. (Zaner R.M. Engelhardt H. T. Jr., Trans.).

[B84] Smith J. A. Fieldsend M. (2021). “Interpretative phenomenological analysis,” in *Qualitative research in psychology: Expanding perspectives in methodology and design*, ed. Camic P. M. (Washington, DC: American Psychological Association), 147–166. 10.1037/0000252-008

[B85] Spinuzzi C. (2003). *Tracing genres through organizations: A sociocultural approach to information design.* Cambridge, MA: MIT Press.

[B86] Swales J. (1996). “Occluded genres in the academy: The case of the submission letter,” in *Academic Writing*, eds Ventola E. Mauranen A. (Corpus Christi TX: Benjamins), 45–58. 10.1075/pbns.41.06swa

[B87] Tardy C. M. (2012). A rhetorical genre theory perspective on L2 writing development. *Writ. Dev.* 2 165–190.

[B88] Thompson E. (2007). *Mind in life: Biology, phenomenology, and the sciences of mind.* Cambridge, MA: Harvard University Press.

[B89] Tomasello M. (2019). *Becoming human: A theory of ontogeny.* Cambridge, MA: Harvard University Press.

[B90] Torday J. S. (2015). Homeostasis as the mechanism of evolution. *Biology* 4 573–590. 10.3390/biology4030573 26389962 PMC4588151

[B91] Turner J. C. Nolen S. B. (2015). Introduction: The relevance of the situative perspective in educational psychology. *Educ. Psychol.* 50 167–172.

[B92] van Manen M. (2017). But is it phenomenology? *Qual. Health Res.* 27 775–779. 10.1177/1049732317699570 28682717

[B93] van Manen M. Adams C. (2009). “The phenomenology of space in writing online,” in *Exploring education through phenomenology: Diverse approaches*, ed. Dall’Alba G. (New York, NY: Wiley), 4–15.

[B94] Varela F. J. (1996). Neurophenomenology: A methodological remedy for the hard problem. *J. Conscious. Stud.* 3 330–349.

[B95] Wardle E. (2009). ‘Mutt Genres’ and the goal of FYC: Can we help students write the genres of the university? *College Compos. Commun.* 60 765–789.

[B96] Williams B. T. (2018). *Literacy practices and perceptions of agency: Composing identities.* London: Routledge.

[B97] Williams J. M. (2011). *Problems into problems: A Rhetoric of Motivation.* Louisville, KY: The WAC Clearinghouse.

[B98] Yañez A. Russell D. R. (2009). “‘The world is too messy’: The challenge of historical literacy in a general education course,” in *Composition(s) in the New Liberal Arts*, eds Post J. C. Inman J. A. (OH: Hampton: Liberty Township), 43–75.

[B99] Yin R. K. (2014). *Case study research: Design and methods*, 5th Edn. Thousand Oaks, CA: Sage Publications.

